# Early changes in apoplast composition associated with defence and disease in interactions between *Phaseolus vulgaris* and the halo blight pathogen *Pseudomonas syringae* Pv. phaseolicola

**DOI:** 10.1111/pce.12770

**Published:** 2016-07-25

**Authors:** Brendan M. O'Leary, Helen C. Neale, Christoph‐Martin Geilfus, Robert W. Jackson, Dawn L. Arnold, Gail M. Preston

**Affiliations:** ^1^ Department of Plant SciencesUniversity of OxfordOxfordOX1 3RBUK; ^2^ Australian Research Council Centre of Excellence in Plant Energy BiologyThe University of Western AustraliaPerth6009Australia; ^3^ Faculty of Health and Applied SciencesUniversity of the West of EnglandBristolBS16 1QYUnited Kingdom; ^4^ Faculty of Agricultural and Nutritional Sciences, Institute of Plant Nutrition and Soil ScienceKiel UniversityKiel24118Germany; ^5^ School of Biological SciencesUniversity of ReadingReadingRG6 6AHUK

**Keywords:** apoplastic washing fluid, citrate, GABA, leaf apoplast, metabolic footprinting, metabolomics, plant defence response, plant–microbe interactions

## Abstract

The apoplast is the arena in which endophytic pathogens such as *Pseudomonas syringae* grow and interact with plant cells. Using metabolomic and ion analysis techniques, this study shows how the composition of *Phaseolus vulgaris* leaf apoplastic fluid changes during the first six hours of compatible and incompatible interactions with two strains of *P. syringae* pv. phaseolicola (*Pph*) that differ in the presence of the genomic island PPHGI‐1. Leaf inoculation with the avirulent island‐carrying strain *Pph* 1302A elicited effector‐triggered immunity (ETI) and resulted in specific changes in apoplast composition, including increases in conductivity, pH, citrate, γ‐aminobutyrate (GABA) and K^+^, that are linked to the onset of plant defence responses. Other apoplastic changes, including increases in Ca^2+^, Fe^2/3+^ Mg^2+^, sucrose, β‐cyanoalanine and several amino acids, occurred to a relatively similar extent in interactions with both *Pph* 1302A and the virulent, island‐less strain *Pph* RJ3. Metabolic footprinting experiments established that *Pph* preferentially metabolizes malate, glucose and glutamate, but excludes certain other abundant apoplastic metabolites, including citrate and GABA, until preferred metabolites are depleted. These results demonstrate that *Pph* is well‐adapted to the leaf apoplast metabolic environment and that loss of PPHGI‐1 enables *Pph* to avoid changes in apoplast composition linked to plant defences.

## Abstract 2

The apoplastic compartment is the arena in which endophytic pathogens such as *Pseudomonas syringae* grow and interact with plant cells. Controlling the composition of the apoplast during plant–pathogen interactions could underlie evolutionary strategies for survival of both the host plant and invading bacteria. Here we combine an apoplast extraction method with metabolomic and ion analyses techniques to describe the composition of the apoplast of *Phaseolus vulgaris* leaves, and to determine changes in apoplast composition associated with resistant and susceptible interactions with the bacterial pathogen *P. syringae* pv. *phaseolicola*. We demonstrate that the apoplast is replete with nutrients that can be used to support pathogen growth, and identify certain ions and metabolites that increases dramatically in the apoplast of plants expressing effector‐triggered immunity

## Introduction

The partially interconnected space between plant cell membranes, known as the apoplast, is a dynamic compartment housing many biological processes including nutrient and water transport, cell wall biosynthesis and defence responses to microbial pathogens (Sattelmacher 2001). The major structural component of the apoplast is the cell wall, which is surrounded by apoplastic fluid and a gaseous phase. Leaf apoplastic fluid contains a variety of proteins, metabolites and inorganic ions whose concentrations are controlled largely by the balance of transport processes with the xylem, phloem and symplasm of surrounding cells, although metabolic processes within the apoplast also contribute to control of metabolite concentrations (Grignon & Sentenac 1991). The composition of leaf apoplastic fluid remains poorly characterized overall but is known to respond to changing physiological conditions including nutrition, abiotic and biotic stress and diurnal cycles (López‐Millán *et al*. 2000a; Sattelmacher 2001). Being one of the first compartments that encounter environmental stress, the apoplast is thought to play an important role in the perception of changing environmental conditions (Hoson 1998).

The apoplast also represents an environmental niche where many plant microbial pathogens establish a parasitic lifestyle. Among the best‐studied occupants of the leaf apoplast are bacteria from the *Pseudomonas syringae* species complex, which gain access to the apoplast through stomata, hydathodes or wounds, and infect a wide variety of plants, causing necrotic symptoms in leaves, stems and fruit. The concentrations of nutrients in tomato, Arabidopsis and tobacco leaf apoplast extracts are sufficient to support rapid growth of *P*. *syringae in vitro* and it has been suggested that *P*. *syringae* strains are metabolically specialized to make efficient use of C and N sources that are abundant in their host's apoplast (Melotto *et al.*, 2008, Rico and Preston, 2008; Rico *et al.*, 2009; Mithani *et al*. 2011). It is not known, however, whether any nutrients can become limiting to bacteria in the apoplast or whether the plant is able to sequester essential nutrients away from the apoplast as a defence mechanism.

Extensive study of *P*. *syringae*–host plant model systems has focused on dissecting the molecular mechanisms and co‐evolution of pathogenesis and plant disease resistance. In particular, *P*. *syringae* use a type‐III secretion system (T3SS) to suppress defence responses by secreting effector proteins directly into host plant cells. Successful suppression of plant defences is essential for the establishment of compatible *P*. *syringae*–host plant interactions which lead to a disease state. However, resistant host plant varieties frequently express resistance proteins (R proteins) that recognize an effector protein or its biological activity and initiate further defence responses to block pathogen multiplication; this defines an incompatible *P*. *syringae*–host plant interaction. In this way, plant defence responses are generally regarded as two tiered (Jones & Dangl 2006). Initial recognition of pathogen associated molecular patterns (PAMPs), e.g. flagellin, elicits PAMP triggered immunity (PTI). Detection of bacterial effector proteins elicits effector triggered immunity (ETI). Effector triggered immunity often culminates in a form of localized programmed cell death known as the hypersensitive response (HR) (Tao *et al.* 2003; Jones & Dangl 2006; Thilmony *et al.* 2006; Truman *et al.* 2006).

The gene‐for‐gene relationships determining infection outcomes between strains of *P*. *syringae* pathovar phaseolicola (*Pph*) and different cultivars of their host plant *Phaseolus vulgaris* (common bean) have been well studied (Taylor *et al*. 1996). The two strains used in this study, *Pph* 1302A and *Pph* RJ3, are directly related, differing only in the presence of a 106 kb genomic island, PPHGI‐1, that can be excised or integrated into the genome *in planta*, thus interconverting the two strains (Jackson *et al*. 2000; Pitman *et al*. 2005; Godfrey *et al*. 2011; Lovell *et al*. 2011). Encoded on this genomic island is an effector gene, *avrPphB* (also called *hopAR1*), whose product induces ETI and the HR in *P*. *vulgaris* cultivar Tendergreen, which encodes the R3 resistance protein (Jackson *et al*. 2000; Shao *et al*. 2003). As a result, these two genetically very similar *Pph* strains differ in their interaction with cultivar Tendergreen plants: *Pph* 1302A contains PPHGI‐1 and elicits the HR; *Pph* RJ3 lacks PPHGI‐1, and establishes a compatible interaction by inhibiting plant defence responses (Jackson *et al*. 2000).

Within the first hours after inoculation of plant leaves with pathogenic bacteria rapid transcriptional, physiological and metabolic changes occur that determine the outcome of this interaction (Jones *et al.* 2006; Thilmony *et al*. 2006; Truman *et al*. 2006; Ward *et al.* 2010; Etalo *et al.* 2013, Yu *et al*. 2013, Mitchell *et al*. 2014). Much of this action is likely to cause rapid changes to conditions within the apoplast, e.g. the downregulation of photosynthesis, production of reactive oxygen species (ROS) and changes in membrane potential and ion fluxes. However, in contrast to whole leaf tissue studies, few studies have examined changes to leaf apoplast composition during infection, and these have mostly focused on proteomic changes (Delaunois *et al*. 2014; Petriccione *et al*. 2014). In this study we employed a robust infiltration–centrifugation apoplastic fluid extraction technique to generate apoplast washing fluid (AWF) samples at early timepoints during compatible and incompatible interactions of *Pph* with *P*. *vulgaris* cultivar Tendergreen leaves. Quantitative analyses of AWF metal ions and polar metabolites, combined with metabolite footprinting experiments that use AWF as a *Pph* growth medium, reveal rapid changes to apoplast composition in both compatible and incompatible interactions that suit the distinct nutritional requirements and metabolic organization of the invading *Pph* strains. Further previously undescribed changes in apoplast metabolite composition were observed to occur following inoculation with the ETI‐eliciting strain 1302A, and prior to any detectable restriction of pathogen growth. We discuss the potential role of these changes in apoplastic processes in the induction and early onset of plant defence responses.

## Results

### The conductance and pH of the *P. vulgaris* leaf apoplast increases following elicitation of ETI

**Figure 1:**
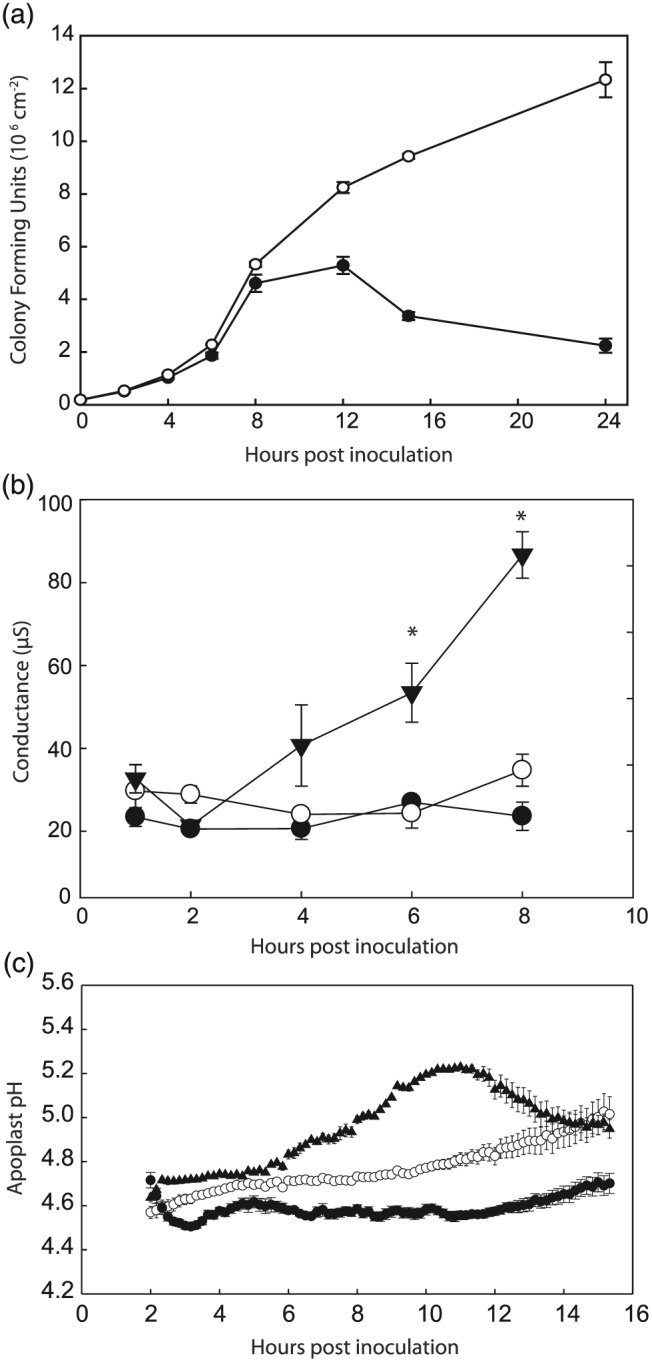
Timing of changes in conductance and pH in *P*. *vulgaris* leaf apoplast during an incompatible interaction. A) Growth of *Pph* 1302A (closed circles) and *Pph* RJ3 (open circles) populations following leaf inoculation. Error bars represent standard error. B) Conductance measurements performed on diluted AWF collected from leaf discs following leaf inoculations with water (closed circles), *Pph* 1302A (closed triangles) and *Pph* RJ3, (open circles). Error bars represent standard error. Asterisks denote a significant difference from control inoculations (ANOVA, *p* < 0.05, *n* = 4). C) Representative in vivo ratiometric pH measurements performed on leaf tissue following inoculation with Oregon green dye and water (closed circles), *Pph* 1302A (closed triangles) and *Pph* RJ3, (open circles). Error bars represent technical standard error calculated by dividing the infected areas into 12 subsections.

It is known that the onset of ETI is associated with changes in membrane transport processes and membrane integrity that may substantially affect apoplast composition. To determine when these changes occurred in our experimental system we observed the time‐dependent progression of Pph interactions with *P. vulgaris* cv. Tendergreen following routine leaf infiltrations with 2 × 10^8^ colony forming units (cfu)/m of Pph RJ3 or Pph 1302A. Leaf inoculation with *Pph* 1302A led to an ETI response, and a clear HR at the site of infection was visible by 24 h post inoculation (hpi; Fig. S1). In contrast, inoculation with *Pph* RJ3 led to a disease state characterized by water soaked lesions visible by 24 hpi. Inoculation of entire leaves with *Pph* 1302A, but not *Pph* RJ3, led to tissue wilting beginning at 8 hpi. Bacterial population size *in planta* was estimated by performing colony counts on homogenized leaf discs (Fig. 1A). Both *Pph* RJ3 and 1302A populations increased similarly for 8 hpi, after which time the *Pph* 1302A population grew more slowly and then declined, presumably because of the onset of ETI (*n* = 3).

Major changes in the composition of infected leaf apoplast are linked to changes in solute movement across adjacent plasma membranes. To estimate when major changes in leaf apoplast composition occurred after inoculations with *Pph*, the conductance of AWF extracted from leaf discs was monitored over time (Fig. 1B). During infection with *Pph* 1302A, the conductance of the AWF increased rapidly between 4 and 8 hpi; this increase preceded the onset of tissue wilting, which subsequently precluded further measurements, as AWF could not be extracted from wilted leaves. With *Pph* RJ3 or water infiltrated leaves, AWF conductance did not change significantly for 8 hpi.

Leaf apoplastic fluid is also known to undergo an increase in pH during defence responses to pathogens (Felle *et al*. 2004, 2005). Here, a non‐destructive microscopy‐based imaging approach using the pH sensitive *F*_490_/*F*_440_ fluorescence ratio of Oregon Green 488‐dextran allowed *in planta* recordings of apoplastic pH for 15 h following infection of *P*. *vulgaris* leaves with *Pph* RJ3 and *Pph* 1302A. Representative timecourse pH measurements are shown in Fig. 1C. Leaf tissue infected with *Pph* 1302A displayed a transient increase in apoplastic pH from 4.8 ± 0.05 to 5.1 ± 0.04 which began approximately 5 hours (301 ± 50 minutes) after inoculation and became most alkaline 8–9 h (530 ± 50 minutes) after inoculation before partially recovering (*n* = 6). An increase in pH during *Pph* RJ3 inoculations occurred later than with *Pph* RJ3 and was not consistently captured by the 15 h experiment (n = 3). In control leaves infiltrated with water, apoplastic pH stablized at 4.6 ± 0.1 during the timecourse (*n* = 3). The observed timing of conductance and pH changes indicated that for subsequent experiments AWF extractions performed at 4 and 6 hpi would be appropriate to capture major changes in apoplast composition following infection with *Pph* 1302A and induction of ETI.

### Characterization of the quality of whole leaf AWF extractions

Quality control assays were established to verify minimal cytoplasmic contamination of AWF extracts from whole *P*. *vulgaris* leaves. All AWF extracts contained detectable protein concentrations and malate dehydrogenase (MDH) activities, which did not differ significantly differ between inoculation treatments (p < 0.05). However, leaves that visibly sustained even slight physical damage during extraction produced AWF with marked increases in both protein concentration and MDH activity that were attributed to cytoplasmic contamination. To discriminate between cytoplasmic contaminated and uncontaminated AWF, a subjective cut‐off was imposed such that only samples with less than 0.25 mg/mL protein and less than 0.4 U/mL MDH activity were considered acceptable for further analyses. The average protein concentration of analysed AWF extracts was 0.18 ± 0.01 mg/mL and the average MDH activity was 0.25 ± 0.02 U/mL (*n* = 64). Glucose‐6‐phosphate was also used as an indicator of cytoplasmic contamination for samples subjected to GC‐MS metabolite analysis. No uncontaminated AWF samples (as determined by the above criteria) contained a detectable signal for glucose‐6‐phosphate except for samples from 6 h infections with *Pph* 1302A wherein five of seven samples contained trace amounts (<1 *μ*M), indicating that plant cell membrane leakiness increased with time during this interaction. The AWF extracts from the 6 h *Pph* 1302A infections were coloured a pale yellow, whereas all other AWF extracts were colourless. Using indigo carmine dye the extent of apoplast dilution caused by infiltration was determined spectrophotometrically to be 2.9 ± 0.2 fold (*n* = 6), and this value was used to correct all concentrations measured from AWF samples.

### AWF composition undergoes specific metabolite and cation changes during early compatible and incompatible interactions

- *GC‐MS metabolite analysis*

**Figure 2:**
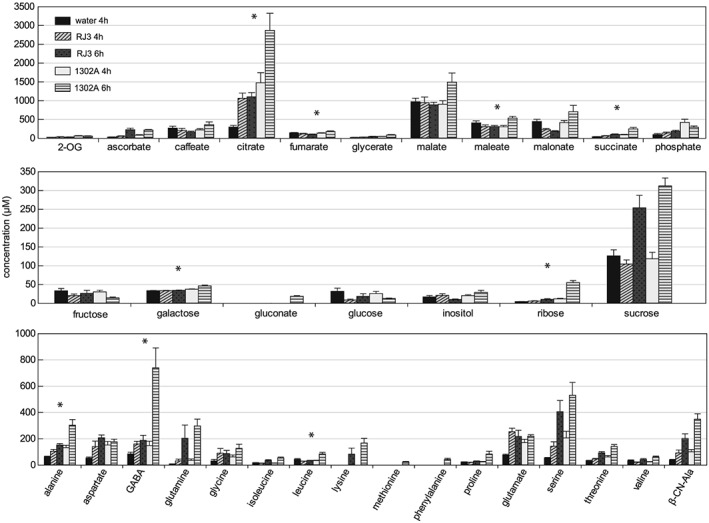
*P*. *vulgaris* leaf AWF metabolite levels determined by GC‐MS at 4 or 6 hpi with water, *Pph* RJ3 or *Pph* 1302A. Metabolites are generally grouped as organic acids, sugars and amino acids in the top, middle and lower panels, respectively. Error bars represent standard error (*n* = 6). Asterisks denote significant differences between *Pph* 1302A and *Pph* RJ3 samples at 6 hpi (ANOVA; *p* < 0.01). Abbreviations: 2‐OG, 2‐oxoglutarate; β‐CN‐Ala, β‐cyanoalanine.

GC‐MS analysis was used to identify and quantify freely soluble, polar metabolites across five AWF sample types: 4 h control inoculations with water, and 4 and 6 h infections with either *Pph* 1302A or *Pph* RJ3 (*n* = 6). Manual inspection of the GC‐MS data identified 60 compound peaks. A further nine major peaks that could not be definitively identified are listed as unknowns with retention times in minutes. Standard curves were generated for 35 identified compounds allowing estimations of the *in planta* apoplastic concentration of these metabolites (Fig. 2, Table S1). Compounds with no standard curve, or where one or more values were below the linear range of quantification, are listed in Table S2 as percentages of the control inoculation signal. The most abundant metabolites detected in the AWF samples were di‐ and tri‐carboxylates: malate, malonate, maleate, citrate and fumarate. Sucrose was the most abundant sugar and GABA was the most abundant amino acid. In total sixteen amino acids were detectable in control AWF; five of these amino acids were non‐proteinogenic, including an unexpected abundance of *β*‐cyanoalanine (37 *μ*M; an intermediate of the plant cyanide detoxification pathway).

**Figure 3:**
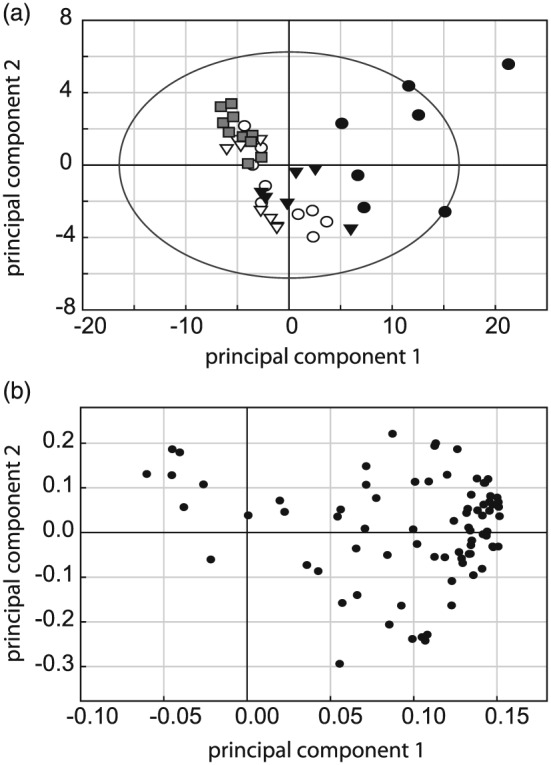
Apoplastic metabolite levels evaluated by PCA show distinct grouping of samples undergoing ETI. A) Scores plot for AWF samples from inoculations with *Pph* 1302A for 4 h (closed triangles) or 6 h (closed circles), *Pph* RJ3 for 4 h (open triangles) or 6 h (open circles) or water for 4 h (grey squares). PC1 and PC2 explain 51.3% and 7.7% of the variation between samples, respectively. The ellipse represents the Hotteling's *T* ^2^ value, 95% confidence limit. B) The corresponding loadings plot shows a clustering of compounds with a positive weight on PC1, indicating that the separation of the 6 hpi *Pph* 1302A samples from control samples along PC1 is due the cumulative contribution of many compounds that have increased in abundance.

The differences in metabolite concentrations in AWF samples following *Pph* infection were remarkable. Principal component analysis revealed that samples from both 6 h infections clearly clustered away from control samples (Fig. 3A). This separation, captured mostly by the first principal component (PC), reflects an increase in most compounds that was generally more severe in *Pph* 1302A infected leaves (Fig. 3B). Nine quantified compounds significantly increased (p < 0.01) in the *Pph* 1302A 6 hpi samples compared to control and *Pph* RJ3 6 hpi samples (Fig. 2, Table S1). In particular, citrate and GABA displayed large absolute increases (from 298 to 2871 *μ*M and 85 to 740 *μ*M, respectively) during the *Pph* 1302A interaction that were not paralleled in the *Pph* RJ3 interaction. Conversely, no compounds significantly increased (p < 0.01) in the *Pph* RJ3 infected leaves compared to *Pph* 1302A infected leaves at either timepoint. Notably, malate was never observed to differ significantly between any treatments. A single unknown compound (unknown 29.466 min; spectrum consistent with a 3‐(hydroxyphenyl)propanoate) significantly decreased upon infection with both *Pph* strains, whereas malonate decreased significantly only during RJ3 infection (Table S2).

*ii*) *AAS of AWF cation concentrations*

**Figure 4:**
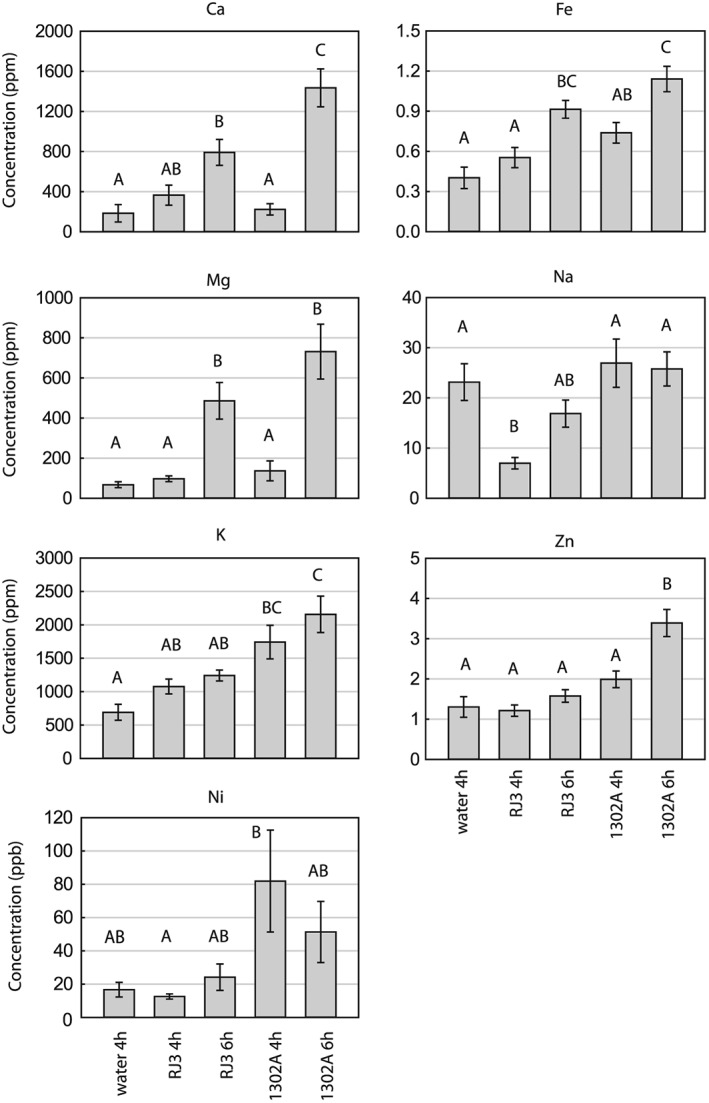
AAS metal ion quantification of *P*. *vulgaris* AWF extracts from healthy and *Pph* 1302A or *Pph* RJ3 infected leaves at 4 or 6 hpi. Error bars represent standard error and letters indicate significant difference between treatments (ANOVA; *p* < 0.05; *n* = 6).

The abundance of metal cations in the apoplast can impact upon plant‐microbe interactions, and K^+^ efflux from the cytosol is a hallmark of plant defence responses (Atkinson *et al*. 1985; Atkinson & Baker 1987; Demidchik *et al*. 2014). To quantify changes in apoplastic metal cation concentrations upon infection, atomic absorption spectroscopy (AAS) for Ca^2+^, K^+^, Mg^2+^, Na^+^, Ni^2+^, Fe^2/3+^, and Zn^2+^ was performed on aliquots from the same AWF extracts used for GC‐MS metabolite analysis (Fig. 4). At 4 hpi, significant (p < 0.05) increases compared to control were only observed for K^+^ and Ni^2+^ within *Pph* 1302A samples (though Ni^2+^ was not significantly increased at 6 hpi). By 6 hpi all other metals except for Na^+^ had significantly increased in the *Pph* 1302A samples. By contrast, in the *Pph* RJ3 samples only Ca^2+^, Fe^2/3+^ and Mg^2+^ significantly increased by 6 hpi. The largest change in absolute concentration was that of K^+^ in the *Pph* 1302A samples, which increased from 18 ± 3 mM in control samples to 55 ± 7 mM by 6 hpi. The detected concentration of K^+^ from healthy leaf apoplast is similar to previous measurements from *Vicia faba* (Mühling & Sattelmacher 1997). Lastly, Ca^2+^ and Mg^2+^ levels were highly correlated across all samples (*R* ^2^ = 0.78).

### Metabolite preferences of Pph in apoplastic fluid mimicking medium

**Figure 5:**
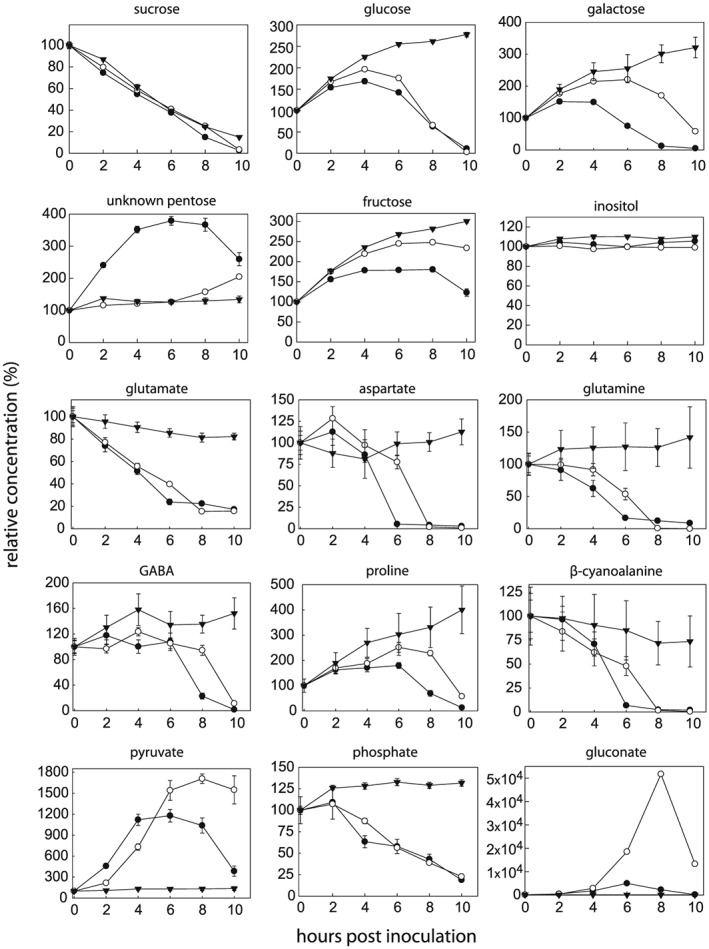
GC‐MS metabolic footprinting reveals the specific nutrient preferences of *Pph* during growth in AWF. Full‐strength AWF from healthy *P*. *vulgaris* leaves was inoculated with *Pph* 1302A (closed circle), *Pph* RJ3 (open circles) or water (closed triangles). Selected metabolite profiles are shown, full data available in Table S3. Error bars represent standard error (*n* = 6).

**Figure 6:**
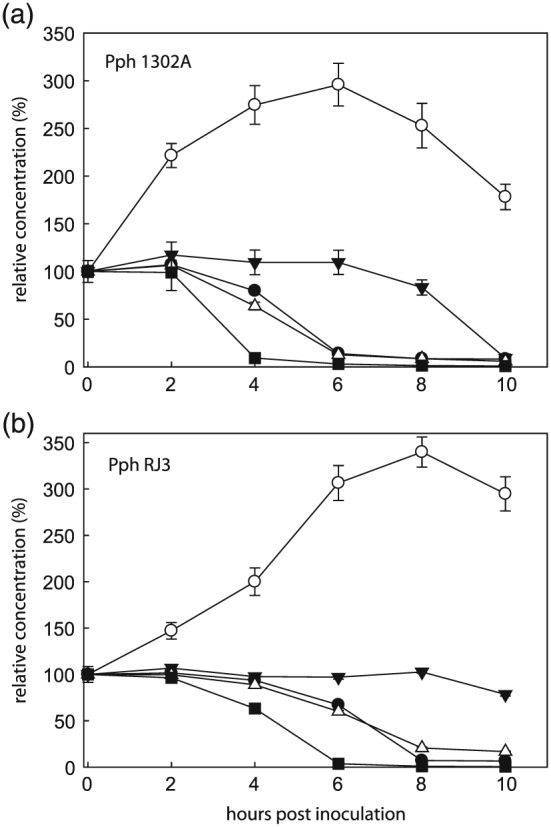
Metabolic footprinting reveals the specific organic acid preferences for *Pph* in apoplast. Full‐strength AWF from healthy *P*. *vulgaris* leaves was inoculated with A) *Pph* 1302A or B) *Pph* RJ3. 2‐oxoglutarate (open circles), citrate (closed triangles), fumarate (close circles), succinate (open triangles) and malate (closed squares). Error bars represent standard error (*n* = 6).

To assess the nutritional preferences of *Pph* 1302A and *Pph* RJ3, a metabolic footprinting timecourse was performed where bacteria initially cultured in M9 minimal medium were incubated in full‐strength *P*. *vulgaris* AWF from healthy plants. Both strains grew equally well in the AWF growth medium (supplemental Fig. 3), which is consistent with the observation that they showed no difference in growth *in planta* during the first 8 hpi (Fig. 1A). Samples of the AWF media were removed every 2 h for 10 h alongside control incubations with un‐inoculated AWF. GC‐MS analyses of the samples identified 61 compounds and an additional 5 unidentified major peaks, most of which changed in abundance during the incubation period (Figs 5, 6, Table S3). The only difference in the direction of metabolism between the two strains was an increase in an unidentified pentose (29.329 min) in the *Pph* 1302A timecourse that did not occur in the RJ3 or control timecourses (Fig. 5). The uniform delay in the onset of metabolite changes in incubations with *Pph* RJ3 compared to 1302A could be because of small differences in the density and/or physiological state of bacteria at the onset of the incubation. Parallel *Pph* 1302A incubations were performed where bacteria were initially cultured in AWF rather than M9 minimal media to observe whether the initial culture conditions affected the metabolic activity of the bacteria in AWF. The patterns of metabolite accumulation and depletion were indistinguishable when *Pph* 1302A was initially cultured in M9 minimal media or AWF (*n* = 5).

Certain metabolite levels, e.g. sucrose and amino acids, changed in the control incubations in the absence of *Pph* and these changes were attributed to the activity of endogenous apoplastic enzymes (Fig. 5, Table S3). Sucrose was depleted in a linear fashion in control and *Pph* incubations. This was likely because of endogenous invertase activity in the AWF and related to concomitant increases in glucose and fructose. Most proteinogenic amino acids increased linearly during the control incubation to varying degrees; the exceptions were glutamate, which decreased in concentration and aspartate and asparagine which did not change. Conversely, among non‐proteinogenic amino acids, only GABA increased in concentration during the control incubation. These patterns indicate protein hydrolysis by proteases and possible GABA formation from glutamate as endogenous enzyme activities in the AWF. Organic acid levels were relatively stable in control incubations with the exception of 2‐oxoglutarate which decreased by 80% over 10 h.

Amid these background changes, *Pph* inoculated samples clearly differed from control samples, and these differences are attributable to bacterial export, uptake and metabolism. Among the various organic acids distinct patterns of accumulation and depletion are apparent (Figs 5 and 6, Table S3). Some dicarboxylates (including dicarboxylic amino acids) were preferred substrates for the bacteria, and were consumed sequentially, with malate and maleate depletion being most rapid, followed by glutamate, aspartate, fumarate and succinate, then citrate and malonate. Interestingly, both 2‐oxoglutarate and pyruvate increased sharply in abundance before beginning to be depleted (Figs 5 and 6). The levels of all amino acids, except homo‐cysteine, decreased during *Pph* incubations compared with control incubations. The rate of depletion of amino acids varied and the bacteria displayed a preference for the uptake and/or catabolism of glutamine, glutamate, aspartate and β‐cyanoalanine (Fig. 5). The hexose sugars glucose and galactose were the predominant carbohydrates metabolized by *Pph*. Sugar alcohols, with the exception of glycerol, were not appreciably metabolized during the incubation (Fig. 5, Table S3). Gluconate, presumably derived from glucose oxidation by bacterial glucose dehydrogenase, displayed by far the largest fold change in abundance of any compound, accumulating greatly in the media for 6 or 8 h before being rapidly consumed. Quantification of the gluconate increase revealed a higher maximum of 1077 ± 16 *μ*M at 8 h for *Pph* RJ3 incubations compared to 216 ± 13 *μ*M at 6 h within *Pph* 1302A incubations (Fig. 5, Table S3).

Carbon substrate utilization profiles were also evaluated for *Pph* 1302A and RJ3 on 95 predetermined metabolites using Biolog GN2 plates (Table S4). These assays determined the capability of *Pph* to oxidize carbon compounds towards the production of reductant, and can therefore infer the presence or absence of metabolic and transport pathways. The preferred compounds for consumption during the metabolite footprinting timecourse, e.g. glucose, dicarboxylates and asparagine, were also all strongly metabolized during the Biolog plate assays (some compounds, e.g. malate and glutamine, are not present in Biolog GN2 plates). Conversely, 2‐oxoglutarate, which was excreted during the metabolite footprinting timecourse, was only weakly utilized in the Biolog assays. Some other compounds, in particular sucrose and inositol, were used when presented as sole C substrates during the Biolog assays but not appreciably metabolized by *Pph* from the AWF media, further indicating the preferential use of certain carbon compounds in the apoplast. Biolog assay results were generally similar for *Pph* 1302A and RJ3, although *Pph* RJ3 tended to display a higher metabolism rate for organic acids, while *Pph* 1302A displayed a higher metabolism rate for several sugars.

## Discussion

### Changes in apoplast composition differ between compatible and incompatible interactions

This study sought to increase knowledge regarding the chemical composition of the leaf apoplast during the early stages of compatible or incompatible bacterial infections. It has previously been established that induction of the HR by a race 1 strain of *Pph* in *P*. *vulgaris* cv. *Red Mexican* requires approximately 4 h, and that marked alteration of cell ultrastructure, which progresses towards cell collapse, is visible beginning at 8 hpi (Roebuck *et al*. 1978). This matches the results reported here, where increases in apoplast conductivity, pH and K^+^ efflux (Figs 1 and 4) commenced near 4–5 hpi in the incompatible interaction, consistent with previous measurements (Atkinson *et al*. 1985; Atkinson & Baker, 1987; Felle *et al*. 2004, 2005), but a negative effect of ETI on bacterial population growth in the leaf was not observed until after 8 hpi. The compatible interaction with *Pph* RJ3 did not cause a similar increase in pH, conductance or K^+^ over the first 6–8 hpi. The delayed and dampened defence response in compatible versus incompatible interactions is well established in the literature (Bonfig *et al*. 2006; Jones & Dangl 2006) and is further confirmed in our metabolomic analyses. The major differences in leaf apoplast composition observed between 4 and 6 hpi with *Pph* 1302A versus RJ3 are therefore most readily attributed to changes in metabolism and membrane transport processes associated with the induction of ETI, rather than direct inhibition of pathogen growth, and include large increases in citrate and GABA. Conversely, changes in apoplast composition that occurred in both compatible and incompatible *Pph* interactions compared to control inoculations are likely to be attributable to bacterial activity and PTI, and include increases in Mg^2+^, Ca^2+^, Fe^2/3+^, β‐cyanoalanine and sucrose as discussed below.

During *Pph* infection of *P*. *vulgaris*, citrate displayed the largest increase in absolute abundance of any metabolite detected and became the most abundant metabolite in AWF. The increase in citrate may reflect an electric charge balancing anion flux from the cytosol to the apoplast accompanying K^+^ efflux, and this is consistent with the increase of several other organic acids in the AWF (Fig. 2). However, in light of recent studies discussed below a further signalling role for apoplastic citrate during bacterial infection is possible and deserves further study. Firstly, *Arabidopsis* leaf discs immersed in 1 mM citrate displayed marked changes in transcript abundance that were not mirrored by immersion in malate, and which most resembled changes associated with biotic stress induction by *P*. *syringae* (Finkemeier *et al*. 2013). Secondly, cytosolic NADP isocitrate dehydrogenase knockouts in *Arabidopsis* that accumulate citrate in their leaves display increased expression of defensive *PR* genes and decreased *P*. *syringae* growth during infection (Mhamdi *et al*. 2010). Thirdly, Anderson *et al*. (2014) observed a dose‐dependent signalling effect of citrate on *P*. *syringae* pv. tomato (*Pto*) DC3000 T3SS gene expression: citrate strongly promoted the expression of the T3SS‐secreted effector protein α‐AvrPto between 50 and 200 *μ*M but completely inhibited expression at 1 mM. These observations may be physiologically important given that apoplastic citrate levels were found in this study to increase from 300 *μ*M in healthy apoplast to greater than 1 mM at 4 hpi with *Pph* (Fig. 2, Table S2).

This study revealed a large increase in the extracellular accumulation of GABA during the incompatible interactions between *P*. *vulgaris* and *Pph*. GABA levels have previously been reported to increase in response to tomato leaf infection by *Cladosporium fulvum* (Solomon & Oliver 2001), and in response to infection of *Arabidopsis thaliana* with *Pto* DC3000 (Ward *et al*. 2010). Although GABA is a potential source of both carbon and nitrogen for *Pph* and other pseudomonads (Rico and Preston 2008), here we show that GABA is not a preferred nutrient (Fig. 5). In plants GABA has long been known to be a stress‐induced metabolite whose accumulation is associated with a range of physiological functions (Shelp *et al*. 2006); and GABA, like citrate, has been shown to suppress *P*. *syringae* T3SS expression when present at high concentrations (Park *et al*. 2010). Nevertheless, the function of extracellular GABA accumulation during the defence response requires elucidation.

### *Metal ion accumulation in the apoplast is associated with* Pph *infection*

There were large correlated increases in Mg^2+^ and Ca^2+^ at 6 hpi in both compatible and incompatible interactions. The dynamic influx of Ca^2+^ from the apoplast to the cytosol via Ca^2+^ channels occurs at the onset of and continues throughout plant defence responses (Grant *et al*. 2000; Felle *et al*. 2004; Shelp *et al*. 2006; Ma *et al*. 2012). These Ca^2+^ influxes cause apoplastic levels of free (unchelated) Ca^2+^ to decrease (Felle *et al*. 2004), while here it was observed that total Ca^2+^ levels increased in AWF extractions following infection. The increase in total Ca^2+^ could be because of release from the vacuole (the main storage compartment for Ca^2+^ in eudicots (Conn *et al*. 2011)) or alternatively could be because of chelation and redistribution of Ca^2+^ by extracellular chelators such as citrate and alginate, a polysaccharide secreted by *P*. *syringae* (Aslam *et al*. 2008). The production of an insoluble material, possibly containing extracellular polysaccharides, was clearly visible by 10 hpi when *Pph* was grown in full‐strength AWF *in vitro*. The chelation of divalent cations would also explain the increase in Mg^2+^ and could support *P*. *syringae* cell wall and biofilm integrity, which require Mg^2+^ and Ca^2+^ (Vaara 1992; Banin *et al*. 2006).

Iron is known to be a major limiting nutrient for *Pseudomonas* in minimal media cultures (Kim *et al*. 2009), and has the potential to be limiting in the apoplast as well. Total iron in the AWF extracts was measured at 7 *μ*M in healthy leaves, which is consistent with previous measurements in sugar beet leaves (López‐Millán *et al*. 2000b). Iron levels increased substantially to 16–20 *μ*M in infected leaves, which are not likely to be limiting for *Pph* growth in the apoplast (Bronstein *et al*. 2008; Kim *et al*. 2009; Jones & Wildermuth 2011).

### Bacterial nutrition is well supported by the composition of the apoplast throughout the early stages of infection

The two metabolomic analyses performed in this study (the quantification of AWF metabolites throughout early infection and the metabolic footprinting experiment using AWF as a *Pph* growth media) present complementary sets of information regarding the status of bacterial nutrition during leaf infection. Metabolic footprinting data indicates that the main substrates fueling *Pph* metabolism are glucose and dicarboxylates (malate, fumarate, succinate, aspartate, glutamate), which remain abundant in AWF extracted throughout early infection. The preferred substrate of *Pph*, malate, is also the most abundant carbon source in healthy apoplast. Thus, although we cannot exclude the possibility that there are differences in the spatial distribution and/or accessibility of apoplastic metabolites *in planta* compared to apoplast extracts, which deserves further investigation, invading *Pph* bacterial populations appear to be replete throughout the early stages of infection with their preferred carbon and nitrogen substrates, and the essential metal cations mentioned above. This result may be affected by the local density of *Pph* and it would be of interest to reproduce these experiments with different inoculum densities.

Several factors may contribute to the sustained availability of apoplastic nutrients throughout the early stages of *Pph* infection. Firstly, reductions in the polarization of the plasma membrane, which form an integral part of defence signalling, would directly increase the availability of many cytosolic substrates to the apoplast. For example the export of organic acids like citrate occurs simultaneously with K^+^ efflux in order to maintain a balance of electric charges (Demidchik *et al*. 2014). Secondly, *Pph* secretes harpin proteins such as HrpZ1 that may function to create pores in the plant plasma membrane, facilitating the introduction of effectors into the cytoplasm and the leakage of cations from the cytosol to the apoplast (Lee *et al*. 2001; Engelhardt *et al*. 2009). Thirdly, as a result of apoplast alkalinisation, transport processes across the plasma membrane may be altered in favour of nutrient accumulation in the apoplast. Atkinson first showed that sucrose efflux to the leaf apoplast increased during infection in a manner related to increased extracellular pH and K^+^ concentration (Atkinson 1987). Fourthly, *Pto* DC3000 infection of Arabidopsis has been reported to cause the upregulation of plasma membrane sugar transport proteins (SWEETs), so it is possible that *P*. *syringae* effectors manipulate sugar efflux to the apoplast (Chen *et al*. 2010, 2012). Lastly, limitation of sucrose and other nutrient export from infected leaves because of callose deposition and activation of extracellular invertases, causes apoplastic accumulation of sugars and other exported metabolites, (Scharte *et al*. 2005). These mechanisms may or may not operate in concert, but one result of *P*. *syringae* infection, as observed here, is a marked increase in apoplastic metabolites such as sucrose, amino acids and organic acids, supporting bacterial nutrition. In future work it would be of interest to systematically determine whether *P*. *syringae* strains deficient in specific effector proteins and/or toxins still elicit the accumulation of apoplastic nutrients.

### *Bacterial glucose*, *α‐keto‐acid and* β‐cyanoalanine *metabolism interfaces with plant defence metabolism*

An increase in apoplastic hexose levels is a well‐known signal promoting heterotrophic respiratory carbon metabolism (Roitsch & González 2004; Bolton 2009). Within infected photosynthesizing leaves a metabolic transition from photosynthesis to respiratory metabolism, stimulated by the inhibition of sucrose export and the activation of hexose producing cell wall invertases, is likely necessary to initiate a full defence response (Scharte *et al*. 2005; Swarbrick *et al*. 2006; Kocal *et al*. 2008; Bonfig *et al*. 2010). It is clear from the disappearance of sucrose in the metabolic footprinting experiments reported here that invertase activity was present in apoplast extracts. However, although sucrose levels increased during both compatible and incompatible interactions, the levels of hexoses (fructose, glucose, galactose) did not show a pronounced increase. Scharte *et al*. (2005) also reported a delay in the accumulation of apoplastic hexoses beyond the accumulation of sucrose and extractable invertase activity. This could be because of rapid and increasing uptake of hexoses by infected leaf cells to fuel respiration and defence compound biosynthesis (Fotopoulos *et al*. 2003), or alternatively because of post‐translational control of apoplast invertases (Bonfig *et al*. 2010).

**Figure 7:**
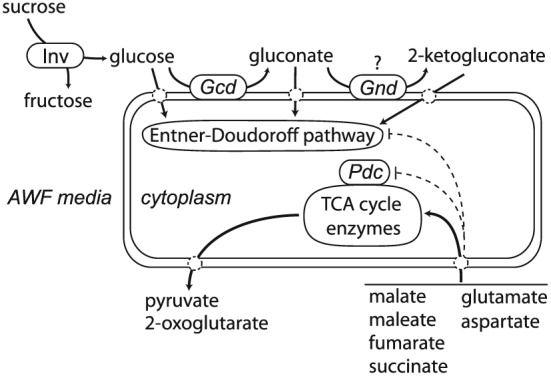
Simplified diagram depicting two major aspects of *Pph* metabolism predicted to be occurring within AWF media based on metabolic footprinting data. In the first pathway, extracellular hydrolysis of sucrose by apoplastic invertases (*Inv*) provides the substrate for the periplasmic conversion of glucose to gluconate by membrane localized glucose dehydrogenase (*Gcd*) which in turn energizes the bacterial electron transport chain. Gluconate may be further oxidized to 2‐ketogluconate. The uptake of glucose, gluconate and 2‐ketogluconate provides substrates for the Entner–Doudoroff pathway. In the second pathway, uptake of preferred dicarboxylic acids provides the major carbon and nitrogen sources for the rapidly growing *Pph* populations. The excretion of pyruvate and 2‐oxoglutarate could represent metabolic endpoints which are because of the regulation of TCA cycle and associated enzymes; e.?g. possible inhibition of the pyruvate dehydrogenase (Pdc) and 2‐oxoglutarate dehydrogenase complexes. Transamination reactions from glutamate to create other amino acids would contribute to the production of 2‐oxoglutarate (not shown). The extracellular accumulation of pyruvate, 2‐oxoglutarate and gluconate appeared to be under the control of catabolite repression by the preferred dicarboxylic acids as indicated by dashed lines.

We observe from the metabolic footprinting experiment that the *Pph* strains studied do not appreciably consume sucrose or alter the rate of sucrose hydrolysis. The bacteria do rapidly produce gluconate, which is most obviously produced from glucose oxidation by the periplasmic bound enzyme glucose dehydrogenase (Fig. 7). Gluconate excretion by *P*. *aeruginosa* into liquid medium has been previously shown and would directly contribute electrons to the bacterial electron transport chain, as would further oxidation of gluconate to 2‐ketogluconate (Behrends *et al*. 2013). Glucose oxidation to gluconate therefore provides a rapid source of energy to *P*. *syringae in planta*.

The metabolic footprinting experiment revealed clear evidence of catabolite suppression in line with what has previously been observed for Pseudomonads: that the dicarboxylic acids repress the uptake and metabolism of sugars by the Entner–Doudoroff pathway (Rojo 2010; Behrends *et al*. 2013). Alleviation of catabolite repression is likely responsible for the sudden reversal of gluconate accumulation in the AWF media that occurred around the same time that preferred dicarboxylic acids were exhausted (Figs 4 and 5; Behrends *et al*. 2013). Furthermore, pyruvate and 2‐oxoglutarate displayed patterns of rapid accumulation and depletion synchronous with those of gluconate. The excretion of pyruvate and 2‐oxoglutarate by pseudomonads has been commonly observed and associated with inhibition of the pyruvate dehydrogenase and 2‐oxoglutarate dehydrogenase complexes (Behrends *et al*. 2013; Lundgren *et al*. 2014). Therefore, we hypothesize that upon exhaustion of preferred organic acid substrates *Pph* undergoes a general re‐organization of metabolic flux that involves increased flux through the Entner‐Doudoroff pathway and initiation of an altered flux through the citric acid cycle (Fig. 7).

As previously shown for *Pph* 1448A (Rico & Preston 2008), *Pph* 1302A and RJ3 contain an authentic frameshift mutation in the gene encoding the C_5_‐dicarboxylic acid transporter (*kgtP*), responsible for 2‐oxoglutarate import. This mutation is consistent with the poor oxidation of 2‐oxoglutarate by *Pph* in Biolog assays and indicates that import rather than metabolism is limiting. Interestingly, extracellular accumulation of pyruvate and 2‐oxoglutarate by Pseudomonads has been implicated in both cyanide (CN) and ROS detoxification as these α‐keto acids react non‐enzymatically with CN to form nitriles and with H_2_O_2_ to form decarboxylation products (Andrae *et al*. 1985; Kunz *et al*. 1998; Mailloux *et al*. 2009). As ROS production is a hallmark of the plant defence response, the benefit to *P*. *syringae* of excreting keto‐acids as additional antioxidants is obvious but remains to be directly tested *in planta*.

Cyanide detoxification by invading bacteria is likely to be an important and understudied aspect of adaptation to the apoplast environment. Ethylene production increases during infection and ETI which produces an equimolar amount of the toxin CN as a byproduct (García *et al*. 2013). Liberation of CN from *P*. *vulgaris* leaves was also observed following wounding (Busch *et al.*, 2010). However, the abundance of the nitrile β‐cyanoalanine in the AWF was unexpected as this non‐proteinogenic amino acid is only known to be formed from the combination of cysteine and CN in the plant mitochondria by β‐cyanoalanine synthase, as the preferred route of CN detoxification in plants (García *et al*. 2013). How a large amount of β‐cyanoalanine accumulates in the apoplast is unclear. β‐cyanoalanine is itself toxic and is further detoxified in plants by a cytosolic nitrile‐hydratase (O'Leary *et al*. 2014a). Intriguingly, however, β‐cyanoalanine is preferentially depleted by *Pph* during incubation in AWF suggesting that *Pph* is able to detoxify β‐cyanoalanine *in planta*. Clearly, further research into the effects of CN and β‐cyanoalanine during bacterial colonization of the apoplast is needed.

### Summary

This study provides a substantial dataset increasing our knowledge of the composition of the plant apoplast. Induction of ETI by *Pph* 1302A results in large changes in *P*. *vulgaris* apoplast composition in comparison to the changes observed in response to *Pph* RJ3, which has evolved to avoid elicitation of ETI through loss of the genomic island PPHGI‐1. Providing mechanistic explanations for the distinctive early changes in the apoplastic metabolome of leaves undergoing defence responses, including large increases in GABA, citrate and β‐cyanoalanine, will be of particular interest to determine the extent to which apoplast composition actively contributes to processes underpinning plant defences. In both interactions it is evident that the apoplast is replete with nutrients that can support pathogen growth and metabolism throughout the early stages of infection. However, *Pph* preferentially assimilates a subset of the metabolites present, despite possessing the metabolic capability to assimilate non‐preferred metabolites, and this could be taken into account in considering resistance strategies targeting pathogen metabolism.

## Materials and Methods

### Plant and bacterial growth conditions

*P*. *vulgaris* cv. Tendergreen seeds were obtained from a commercial distributor (Chiltern Seeds). Seeds were sown on moist paper towels and, once germinated, transferred to soil pots (John Innes number 1 compost) and placed in a growth chamber with 18 h of light at 20 °C and 6 h of dark at 18 °C.

For inoculation into leaves, cultures of *Pph* were grown overnight in King's Broth media (KB), pelleted and resuspended in sterile distilled water to an OD_600_ of 0.15 (~2 × 10^8^ CFU/mL). Inoculations were performed by piercing the leaf surface with a fine point and using a 1 mL needleless syringe to infiltrate the entire apoplastic air‐space with the bacterial suspension.

For *in planta* growth assays, a 1 cm diameter cork borer was used to harvest the inoculated leaf tissue. This tissue was homogenized in sterile distilled water, and following centrifugation for 10 s at 1300 rpm the supernatant was serially diluted and spread onto KB plates. For *in vitro* growth assays, diluted overnight culture was added to 200 *μ*L of full strength AWF in a 96‐well plate. Plates were incubated at 25 °C while shaking at 100 rpm. A 10 *μ*L sample was taken at 0, 2, 4, 6, 8 and 24 h, serially diluted and spread onto KB plates. Total CFUs were calculated after incubation of the KB plates for 48 h at 25 °C.

### Leaf apoplast extractions

AWF was extracted from the first true leaves of *P*. *vulgaris* cv. Tendergreen 3–4 weeks post germination and between 8 and 11 h into the light period using ddH_2_O as the infiltration medium as previously described (O'Leary *et al*. 2014b). Biological replicates of AWF samples (indicated as *n* for each analysis) were collected over several days from one batch of plants grown under common conditions. To calculate the dilution of apoplast components during AWF extraction, parallel AWF extractions were performed with and without 50 *μ*M indigo carmine in the infiltration fluid. Ratios of absorbance at A_610_ were used to calculate the apoplast dilution factor as described previously (O'Leary *et al*. 2014b). To approximate *in vivo* solute concentrations ‘full‐strength’ AWF samples were generated by freeze‐drying pooled AWF samples then redissolving the material in distilled water to a volume determined by the apoplast dilution factor. Full‐strength AWF samples were stored at −80 °C.

Malate dehydrogenase activity was measured by monitoring NADH oxidation at A_340_ in 200 *μ*L reactions containing 100 mM HEPES pH 7, 10% glycerol, 0.15 mM NADH, 2 mM oxaloacetate and 20 *μ*L of AWF. Protein concentrations were quantified using the Bio‐Rad Protein Assay Reagent (Bio‐Rad Laboratories) according to the manufacturer's instructions using bovine serum albumin as the protein standard.

### Metabolic footprinting and carbon utilization assays

Metabolic footprinting timecourse incubations of *Pph* in full‐strength AWF were performed using cultures grown overnight in M9 minimal media (Sambrook *et al*. 1989) or in AWF, both to mid log phase. Overnight cultures were pelleted and resuspended in distilled water to an OD_600_ of 0.15. Either 50 *μ*L of diluted culture or water was added to 1 mL of full‐strength AWF from healthy leaves and incubated in a 2 mL tube while shaking at 28 °C. At least five replicate AWF cultures were established for each bacterial strain and 150 *μ*L aliquots were removed at 0, 2, 4, 6, 8 and 10 hpi and centrifuged at 10 000 *g* for 5 min to pellet the bacteria. The supernatant was removed, frozen in liquid N_2_ and stored at −80 °C before analysis by GC‐MS. Carbon utilization assays for *Pph* 1302A and RJ3 were performed using GN2 Biolog plates (Hayward, USA) as described previously (Rico & Preston 2008).

### Conductance assays

For measurements of conductance, AWF was extracted from *P*. *vulgaris* cv. Tendergreen leaf discs obtained using a 2 cm diameter cork borer. After rinsing and infiltration of the leaf discs with ddH_2_O, excess surface liquid was removed with a paper towel. Each leaf disc was then inserted into a cut 1 mL pipette tip which was inserted into a 1.5 mL tube and centrifuged at 600 g for 5 min. The eluted AWF was centrifuged again at 17 000 *g* for 2 min, then 25 *μ*L of supernatant was then diluted to 3 mL with ddH_2_O and the conductance measured using a conductivity meter (Jenway).

### GC‐MS analysis

Samples were prepared for GC‐MS analysis using a modified version of the method of Lisec *et al* (2006). Frozen 150 *μ*L samples of AWF or cell‐free AWF media from bacterial culture were extracted in 700 *μ*L of methanol containing 10 *μ*g/mL of ribitol while shaking at 70 °C for 10 min, followed by centrifugation for 5 min at 11 000 *g*. Next, 700 *μ*L of supernatant was removed and mixed sequentially by vortexing with 375 *μ*L of cold chloroform, then 500 *μ*L of ddH_2_O. Samples were centrifuged at 2200 *g* for 15 min, then 150 *μ*L of supernatant was transferred to a fresh tube and dried in a vacuum concentrator without heat. The samples were derivitized in 29 *μ*L of pyridine containing 20 mg/mL methoxyamine and 50 *μ*L of N‐Methyl‐N‐(trimethylsilyl)trifluoroaceamide (MSTFA) as described previously (Lisec *et al.* 2006). GC‐MS analysis was performed as described previously (O'Leary *et al*. 2014a). For absolute quantification of metabolites standard curves were generated from pure stock solutions that were extracted and derived as above. Multivariate analyses were performed with SIMCA software (Umetrics), with data mean‐centred and scaled to unit variance.

### Metal cation analysis

Metal cation concentrations within AWF were determined using a Beckman AAS equipped with graphite furnace in the case of Ni, Ca, Mg, Fe, Na, and Zn or an acetylene flame for K. Standard curves were created for each element and AWF samples were diluted in ddH_2_O to be within the linear range.

### Ratiometric quantification of apoplastic pH

For *in planta* quantitation of leaf apoplastic pH, 25 *μ*M of the ratiometric fluorescent pH indicator dye Oregon Green 488‐dextran (Invitrogen GmbH, Darmstadt, Germany) was loaded with or without 2 × 10^8^ CFU/mL of *Pph* into the leaf apoplast of intact plants using a needleless syringe. The 10 kDa dextran conjugation ensures that the dye does not access the cytoplasm from the apoplastic space (Geilfus & Mühling 2012). As established previously, all pH responses were monitored starting 2 hpi to ensure evaporation of excess infiltrated water and normal gas exchange within the apoplast (Geilfus & Mühling 2011). Fluorescence images at excitation wavelengths 440/20 and 490/10 nm were collected with a Leica inverted microscope via 50‐fold magnification (0.15 numerical aperture, dry objective; voxel size = 0.002 mm; HCX PL FLUOTAR L, Leica Microsystems) with the optical and instrumental settings described elsewhere (Geilfus & Mühling 2011). Exposure time was 75 ms for both channels. The fluorescence ratio *F*_490_/*F*_440_ was obtained as a measurement of pH on a pixel‐by‐pixel basis using LAS AF software (version 2.3.5; Leica Microsystems). Background values were subtracted at each channel. For conversion of the fluorescence ratio data into apoplastic pH, an *in vivo* calibration was conducted (Fig. S2).

## Supporting information


**Figure S1**: Infection of *P*. *vulgaris* leaves with *Pph* 1302A and *Pph* RJ3 leads to an incompatible and compatible interaction, respectively.
**Figure S2**: *In vivo c*alibration of the ratiometric pH measurements.
**Figure S3:** Comparison of i*n vitro* population growth of *Pph* 1302A versus RJ3 in full strength AWF.
**Table S1**: GC‐MS determined absolute concentrations of compounds in leaf AWF extractions.
**Table S2**: GC‐MS determined relative concentrations of compounds in leaf AWF extractions.
**Table S3**: Values for metabolic footprinting analysis of *Pph* incubated in AWF.
**Table S4**: Values for carbon utilization assays of *Pph* performed on GN2 Biolog plates.

Figure S1 Supporting info item

Table S1 Supporting info item
