# Supplementary material for: Early changes in apoplast composition associated with defence and disease in interactions between Phaseolus vulgaris and the halo blight pathogen Pseudomonas syringae Pv. phaseolicola
Source: Plant Cell Environ. 2016 Jul 25;39(10):2172–84. doi: 10.1111/pce.12770 (PMC5026161; doi:10.1111/pce.12770)
Supplement: Supplementary file 1 — Figure S1 Supporting info item [file PCE-39-2172-s001.docx]

**Supporting Information**

**Figure S1:** Infection of *P. vulgaris* leaves with *Pph* 1302A and *Pph* RJ3 leads to an incompatible and compatible interaction respectively.

**Figure S2:** *In vivo c*alibration of the ratiometric pH measurements.

**Figure S3:** Comparison of i*n vitro* population growth of *Pph* 1302A versus RJ3 in full strength AWF.

**Table S1:** GC-MS determined absolute concentrations of compounds in leaf AWF extractions.

**Table S2:** GC-MS determined relative concentrations of compounds in leaf AWF extractions.

**Supplemental Figure 1**

**
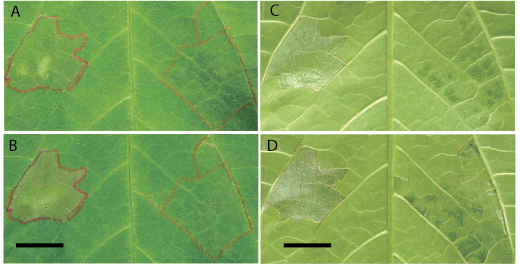
**

**Supplemental Figure 1:** Infection of *Phaseolus vulgaris* cv. Tendergreen leaves with *Pph* 1302A and *Pph* RJ3 leads to an incompatible (resistance) andcompatible (disease) interaction, respectively. The adaxial (A-B) and abaxial (C-D) surface of *P. vulgaris* leaves were photographed 24 (A,C) and 48 hpi (B,D). The inoculated tissue area is outlined on the adaxial surface. The left inoculation in each panel is a representative *Pph* 1302A infection triggering an HR and the right inoculation is representative of a *Pph* RJ3 infection causing water soaked lesions. The scale bars are 10 mm long.

**Supplemental Figure 2**

**
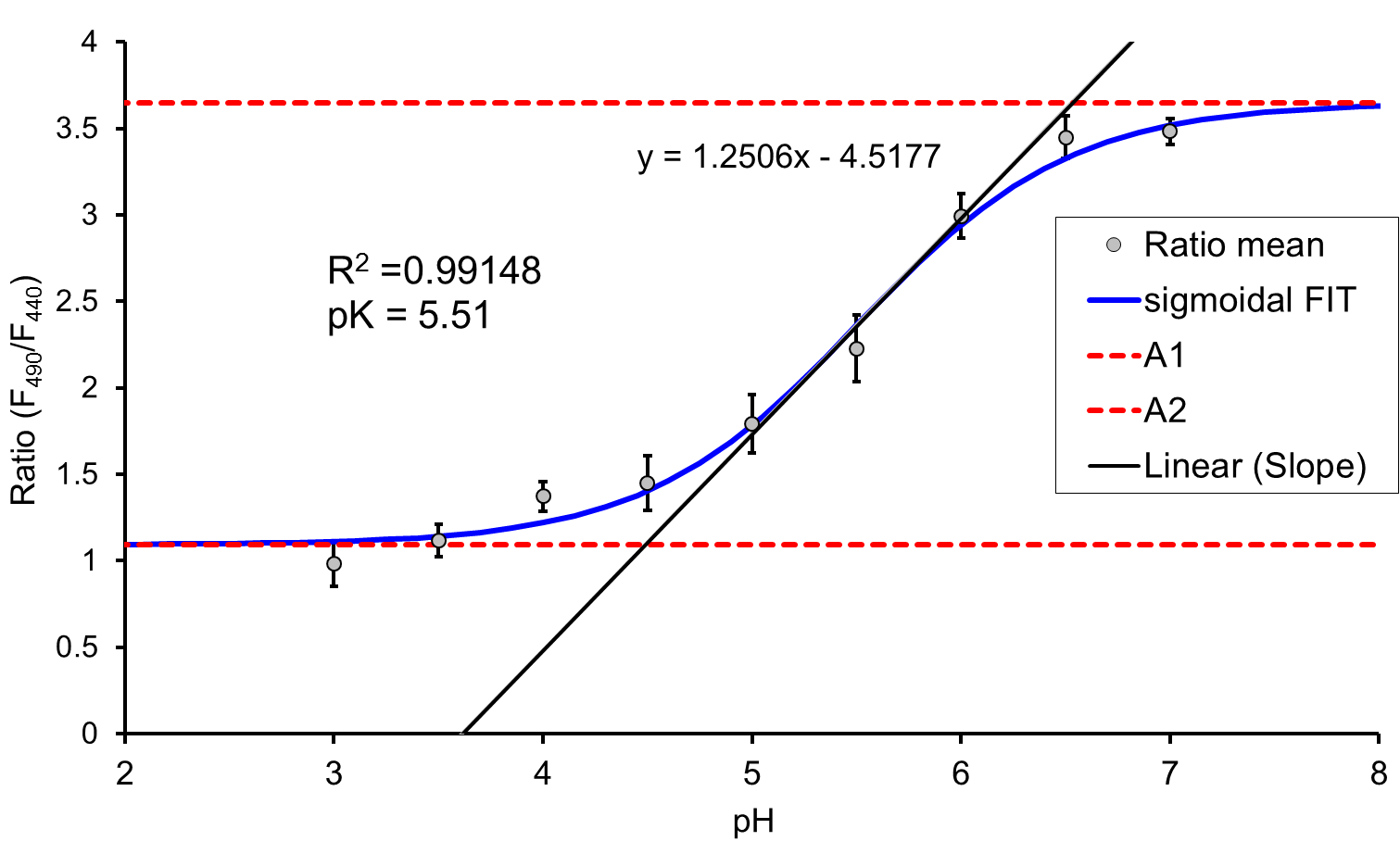
**

**Supplemental Figure 2:** *In vivo c*alibration of the ratiometric pH measurements.

Oregon Green dye solutions were pH buffered with citric acid/sodium citrate (3.0 ≤ pH ≤ 5.0; 10 mM), MES (5.5 ≤ pH ≤ 6.0; 50 mM) and PIPES (6.5 ≤ pH ≤ 7.5; 50 mM) and were subsequently loaded into the leaf apoplast as described in materials and methods. The Boltzmann fit was chosen to fit sigmoidal curves to the calibration. Fitting yielded an area of best responsiveness in the range pH 3.8 –6.4.

**Supplemental Figure 3**


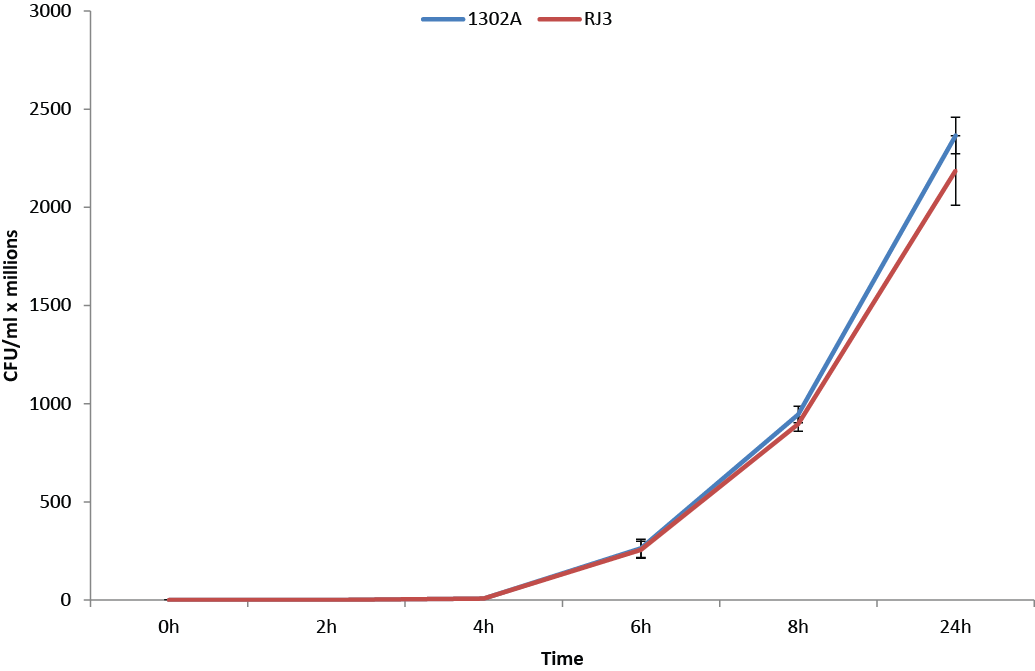


**Supplemental Figure 3:** Comparison of i*n vitro* population growth of *Pph* 1302A versus RJ3 in full strength AWF. Error bars represent standard error, n=4.

**Supplemental Table 1:** Concentrations of compounds in leaf AWF extracted at 4 or 6 hpi from *P. vulgaris* leaves inoculated with water (control), *Pph* 1302A or *Pph* RJ3 (n=6).

| **Metabolite** | **Group** | **Control**  **(µm)** | | | **RJ3 4hpi**  **(µm)** | | | **RJ3 6hpi**  **(µm)** | | | **1302A 4hpi**  **(µm)** | | | **1302A 6hpi**  **(µm)** | | |
| --- | --- | --- | --- | --- | --- | --- | --- | --- | --- | --- | --- | --- | --- | --- | --- | --- |
| malate | A | 972 | ± | 93 | 948 | ± | 149 | 889 | ± | 68 | 907 | ± | 94 | 1493 | ± | 244 |
| malonate | B | 448 | ± | 56 | 232 | ± | 30 | 184 | ± | 19 | 417 | ± | 59 | 710 | ± | 166 |
| maleate | A† | 411 | ± | 53 | 310 | ± | 46 | 310 | ± | 32 | 306 | ± | 36 | 537 | ± | 48 |
| citrate | C† | 298 | ± | 44 | 1063 | ± | 141 | 1101 | ± | 114 | 1477 | ± | 268 | 2871 | ± | 454 |
| *trans*-caffeate | A | 271 | ± | 50 | 215 | ± | 51 | 162 | ± | 25 | 226 | ± | 31 | 363 | ± | 72 |
| fumarate | D† | 145 | ± | 10 | 118 | ± | 14 | 103 | ± | 11 | 137 | ± | 12 | 180 | ± | 17 |
| sucrose | C | 126 | ± | 16 | 105 | ± | 11 | 254 | ± | 33 | 118 | ± | 18 | 312 | ± | 21 |
| phosphate | D | 96 | ± | 29 | 141 | ± | 26 | 187 | ± | 28 | 424 | ± | 82 | 292 | ± | 35 |
| GABA | C† | 85 | ± | 12 | 160 | ± | 20 | 188 | ± | 38 | 149 | ± | 33 | 740 | ± | 151 |
| glutamate | C | 77 | ± | 7 | 254 | ± | 27 | 217 | ± | 48 | 171 | ± | 25 | 219 | ± | 12 |
| alanine | C† | 65 | ± | 3 | 105 | ± | 13 | 153 | ± | 11 | 133 | ± | 18 | 303 | ± | 43 |
| serine | C | 54 | ± | 4 | 143 | ± | 35 | 406 | ± | 85 | 207 | ± | 51 | 530 | ± | 98 |
| aspartate | C | 53 | ± | 8 | 140 | ± | 42 | 208 | ± | 21 | 152 | ± | 28 | 177 | ± | 20 |
| galactose | D† | 34 | ± | 1 | 34 | ± | 1 | 35 | ± | 1 | 38 | ± | 1 | 46 | ± | 2† |
| leucine | D† | 43 | ± | 6 | 30 | ± | 1 | 33 | ± | 2 | 34 | ± | 2 | 85 | ± | 12 |
| succinate | C† | 42 | ± | 5 | 63 | ± | 8 | 104 | ± | 11 | 95 | ± | 11 | 251 | ± | 38 |
| β-cyanoalanine | C | 38 | ± | 6 | 92 | ± | 22 | 202 | ± | 36 | 101 | ± | 17 | 349 | ± | 42 |
| threonine | C | 35 | ± | 1 | 45 | ± | 6 | 93 | ± | 9 | 62 | ± | 10 | 142 | ± | 14 |
| dehydroascorbate | C | 34 | ± | 5 | 56 | ± | 12 | 227 | ± | 42 | 84 | ± | 12 | 220 | ± | 14 |
| valine | D | 34 | ± | 5 | 22 | ± | 2 | 42 | ± | 7 | 25 | ± | 3 | 62 | ± | 7 |
| fructose | A | 33 | ± | 6 | 20 | ± | 4 | 27 | ± | 8 | 30 | ± | 4 | 15 | ± | 2 |
| glucose | A | 32 | ± | 8 | 9 | ± | 2 | 19 | ± | 7 | 26 | ± | 6 | 13 | ± | 1 |
| glycine | A | 32 | ± | 11 | 91 | ± | 35 | 87 | ± | 24 | 62 | ± | 15 | 127 | ± | 31 |
| phenylalanine |  | n.d. <28 | | | n.d. <28 | | | n.d. <28 | | | n.d. <28 | | | 42 | ± | 9 |
| 2-oxoglutarate | D | 25 | ± | 2 | 37 | ± | 8 | 34 | ± | 4 | 62 | ± | 11 | 58 | ± | 9 |
| lysine |  | n.d. <25 | | | n.d. <25 | | | 83 | ± | 46 | n.d. <25 | | | 168 | ± | 34 |
| proline | D | 23 | ± | 1 | 20 | ± | 2 | 30 | ± | 3 | 25 | ± | 3 | 85 | ± | 20 |
| glycerate | C | 22 | ± | 3 | 31 | ± | 4 | 49 | ± | 7 | 51 | ± | 5 | 88 | ± | 12 |
| isoleucine | C | 17 | ± | 1 | 14 | ± | 2 | 35 | ± | 6 | 15 | ± | 1 | 54 | ± | 7 |
| inositol | A | 17 | ± | 4 | 21 | ± | 4 | 10 | ± | 1 | 20 | ± | 2 | 29 | ± | 6 |
| methionine |  | n.d. <13 | | | n.d. <13 | | | n.d. <13 | | | n.d. <13 | | | 24 | ± | 5 |
| gluconate |  | n.d. <8 | | | n.d. <8 | | | n.d. <8 | | | n.d. <8 | | | 19 | ± | 2 |
| glutamine | C | 7 | ± | 2 | 32 | ± | 13 | 204 | ± | 101 | 36 | ± | 11 | 298 | ± | 52 |
| ribose | C† | 5 | ± | 1 | 6 | ± | 1 | 11 | ± | 2 | 12 | ± | 2 | 55 | ± | 5 |
| nicotinate | D | 2 | ± | 1 | 4 | ± | 1 | 4 | ± | 1 | 10 | ± | 1 | 6 | ± | 1 |

A: metabolite concentration did not significantly change during any incubation compared to control (*p*>0.01; ANOVA)

B: metabolite significantly decreased during RJ3 infection only (*p*<0.01; ANOVA)

C: metabolite significantly increased during both Pph RJ3 and 1302A incubations (*p*<0.01; ANOVA)

D: metabolite significantly increased during Pph 1302A infection only (*p*<0.01; ANOVA)

† Indicates a significant difference between the 6h incubation with *Pph­* 1302A and RJ3 (t-test; p<0.01)

**Supplemental Table 2:** Relative abundances of compounds not absolutely quantifiable in AWF extracted at 4 or 6 hpi from leaves inoculated with water (control), *Pph* 1302A or *Pph* RJ3 (n=6). Values are presented as a percentage of control with standard error. Note, these metabolite signals may not be in the linear range.

| **Metabolite** | **Group** | | **Control** | | | **RJ3 4hpi** | | | **RJ3 6hpi** | | | **1302A 4hpi** | | | **1302A 6hpi** | | | |
| --- | --- | --- | --- | --- | --- | --- | --- | --- | --- | --- | --- | --- | --- | --- | --- | --- | --- | --- |
| aconitate | | C† | 100 | ± | 21 | 286 | ± | 55 | 323 | ± | 31 | 482 | ± | 81 | 853 | ± | 119 |  |
| asparagine | | D† | 100 | ± | 27 | 71 | ± | 30 | 94 | ± | 40 | 67 | ± | 33 | 2233 | ± | 1047 |  |
| β-alanine | | C | 100 | ± | 25 | 316 | ± | 56 | 363 | ± | 71 | 327 | ± | 81 | 1014 | ± | 220 |  |
| *cis-*caffeate | | A | 100 | ± | 17 | 119 | ± | 32 | 185 | ± | 26 | 67 | ± | 8 | 121 | ± | 24 |  |
| cis-*p*-coumarate | | D† | 100 | ± | 24 | 167 | ± | 39 | 156 | ± | 36 | 240 | ± | 41 | 365 | ± | 47 |  |
| ethanolamine | | C† | 100 | ± | 13 | 225 | ± | 30 | 394 | ± | 55 | 220 | ± | 31 | 1138 | ± | 193 |  |
| galactonate | | C† | 100 | ± | 13 | 130 | ± | 13 | 171 | ± | 18 | 158 | ± | 23 | 464 | ± | 44 |  |
| gluconate | | C | 100 | ± | 51 | 370 | ± | 152 | 1446 | ± | 560 | 756 | ± | 219 | 3761 | ± | 729 |  |
| gluconate-1-4-lactone | | C | 100 | ± | 4 | 227 | ± | 19 | 212 | ± | 14 | 283 | ± | 33 | 309 | ± | 38 |  |
| glycerol | | D† | 100 | ± | 8 | 76 | ± | 4 | 86 | ± | 7 | 116 | ± | 9 | 151 | ± | 12 |  |
| homocysteine | | A | 100 | ± | 16 | 79 | ± | 29 | 450 | ± | 226 | 211 | ± | 46 | 300 | ± | 61 |  |
| lactate | | D | 100 | ± | 15 | 104 | ± | 14 | 136 | ± | 39 | 119 | ± | 19 | 258 | ± | 23 |  |
| lysine | | C | 100 | ± | 40 | 1201 | ± | 435 | 10430 | ± | 3332 | 2183 | ± | 937 | 16562 | ± | 2488 |  |
| maleamate | | D† | 100 | ± | 16 | 49 | ± | 14 | 69 | ± | 26 | 424 | ± | 83 | 358 | ± | 53 |  |
| mannitol | | C | 100 | ± | 4 | 158 | ± | 9 | 247 | ± | 21 | 182 | ± | 14 | 303 | ± | 28 |  |
| methionine | | D | 100 | ± | 100 | 303 | ± | 303 | 2596 | ± | 1155 | 697 | ± | 278 | 5164 | ± | 970 |  |
| phenylalanine | | D | 100 | ± | 27 | 562 | ± | 236 | 2744 | ± | 970 | 955 | ± | 304 | 8620 | ± | 2079 |  |
| putrescine | | D† | 100 | ± | 86 | 665 | ± | 300 | 1396 | ± | 468 | 171 | ± | 86 | 14297 | ± | 2318 |  |
| pyruvate | | C | 100 | ± | 19 | 463 | ± | 137 | 1488 | ± | 595 | 772 | ± | 218 | 2138 | ± | 375 |  |
| salicylate | | A | 100 | ± | 4 | 166 | ± | 48 | 57 | ± | 13 | 204 | ± | 79 | 114 | ± | 11 |  |
| sorbitol | | A | 100 | ± | 6 | 166 | ± | 46 | 167 | ± | 23 | 152 | ± | 20 | 162 | ± | 9 |  |
| threitol | | C | 100 | ± | 24 | 138 | ± | 46 | 379 | ± | 82 | 216 | ± | 46 | 627 | ± | 159 |  |
| *trans*-*p*-coumarate | | D† | 100 | ± | 22 | 135 | ± | 33 | 107 | ± | 29 | 244 | ± | 35 | 281 | ± | 32 |  |
| tyrosine | | D | 100 | ± | 29 | 88 | ± | 32 | 560 | ± | 318 | 200 | ± | 79 | 2364 | ± | 782 |  |
| unknown 22.948 | | A | 100 | ± | 2 | 108 | ± | 2 | 106 | ± | 1 | 118 | ± | 9 | 114 | ± | 11 |  |
| unknown 29.466 | | E† | 100 | ± | 6 | 58 | ± | 8 | 28 | ± | 3 | 96 | ± | 7 | 58 | ± | 7 |  |
| unknown 30.279 | | D | 100 | ± | 14 | 167 | ± | 23 | 160 | ± | 16 | 216 | ± | 22 | 252 | ± | 36 |  |
| unknown 30.855 | | C | 100 | ± | 15 | 193 | ± | 30 | 220 | ± | 24 | 209 | ± | 19 | 287 | ± | 41 |  |
| unknown 35.139 | | C† | 100 | ± | 17 | 126 | ± | 17 | 248 | ± | 18 | 248 | ± | 53 | 662 | ± | 49 |  |
| unknown 35.827 | | A | 100 | ± | 6 | 90 | ± | 7 | 90 | ± | 8 | 122 | ± | 13 | 108 | ± | 26 |  |
| unknown 37.6 | | C† | 100 | ± | 15 | 135 | ± | 15 | 263 | ± | 22 | 165 | ± | 22 | 500 | ± | 38 |  |
| unknown 47.731 | | D | 100 | ± | 29 | 123 | ± | 57 | 5500 | ± | 2737 | 575 | ± | 270 | 16138 | ± | 2408 |  |
| unknown 48.113 | | D | 100 | ± | 26 | 75 | ± | 44 | 5797 | ± | 3581 | 480 | ± | 199 | 15942 | ± | 2403 |  |
| urea | | D† | 100 | ± | 11 | 110 | ± | 25 | 173 | ± | 31 | 176 | ± | 36 | 473 | ± | 61 |  |
| xylitol | | C† | 100 | ± | 9 | 177 | ± | 13 | 298 | ± | 15 | 215 | ± | 24 | 584 | ± | 61 |  |
| xylonate | | C† | 100 | ± | 18 | 136 | ± | 16 | 202 | ± | 18 | 159 | ± | 23 | 627 | ± | 66 |  |
| xylose | | D† | 100 | ± | 8 | 126 | ± | 17 | 118 | ± | 16 | 177 | ± | 20 | 306 | ± | 53 |  |
| xylulose | | D | 100 | ± | 36 | 29 | ± | 6 | 200 | ± | 80 | 30 | ± | 6 | 706 | ± | 154 |  |

A: metabolite concentration did not significantly change during any incubation compared to control (*p*>0.01; ANOVA)

C: metabolite significantly increased during both Pph RJ3 and 1302A incubations (*p*<0.01; ANOVA)

D: metabolite significantly increased only during Pph 1302A infection (*p*<0.01; ANOVA)

E: metabolite significantly decreased during both Pph RJ3 and 1302A infections (*p*<0.01; ANOVA)

† Indicates a significant difference between the 6h incubation with *Pph­* 1302A and RJ3 (t-test; p<0.01)
